# Indirect genetic effects allow escape from the inefficient equilibrium in a coordination game

**DOI:** 10.1002/evl3.155

**Published:** 2020-05-01

**Authors:** Arthur Bernard, Nicolas Bredeche, Jean‐Baptiste André

**Affiliations:** ^1^ Sorbonne Université, CNRS Institut des Systèmes Intelligents et de Robotique F‐75005 Paris France; ^2^ Department of Ecology and Evolution University of Lausanne CH‐1015 Lausanne Switzerland; ^3^ Institut Jean Nicod, Département d'études cognitives, ENS, EHESS PSL Research University, CNRS Paris France

**Keywords:** Collective action, evolution of cooperation, evolutionary robotics, evolutionary game theory, equilibrium selection, plasticity

## Abstract

Social interactions involving coordination between individuals are subject to an “evolutionary trap.” Once a suboptimal strategy has evolved, mutants playing an alternative strategy are counterselected because they fail to coordinate with the majority. This creates a detrimental situation from which evolution cannot escape, preventing the evolution of efficient collective behaviors. Here, we study this problem using evolutionary robotics simulations. We first confirm the existence of an evolutionary trap in a simple setting. We then, however, reveal that evolution can solve this problem in a more realistic setting where individuals need to coordinate with one another. In this setting, simulated robots evolve an ability to adapt plastically their behavior to one another, as this improves the efficiency of their interaction. This ability has an unintended evolutionary consequence: a genetic mutation affecting one individual's behavior also indirectly alters their partner's behavior because the two individuals influence one another. As a consequence of this indirect genetic effect, pairs of partners can virtually change strategy together with a single mutation, and the evolutionary barrier between alternative strategies disappears. This finding reveals a general principle that could play a role in nature to smoothen the transition to efficient collective behaviors in all games with multiple equilibriums.

The success of a collective action often hinges on the coordinated decisions of several individuals. For instance, carrying out a collective hunt implies that all individuals hunt at the same time, agree on a common prey, and pursue the prey in a coordinated manner. Thus, collective efficiency does not depend solely on the skills of a single individual but emerges from the ability of the group to act together (Alvard and Nolin 2002; Alvard 2003; Drea and Carter 2009). This raises the question of how natural selection, which acts on individuals, can shape such collective behaviors.

This problem can be formalized with a specific class of games called “coordination games.” To understand, let us consider a situation in which two hunters must coordinate to capture a prey, but have to make a choice between a prey with a high nutritive value and a prey with a low nutritive value. The strategy of choosing the most nutritious prey is evolutionarily stable. If everyone chooses this prey, one's best response is to choose this prey as well. But choosing the poorly nutritious prey is also evolutionarily stable. If everyone chooses the poorly nutritious prey, there is nothing better one can do than choose the same. The existence of this second, suboptimal, evolutionarily stable strategy (ESS) raises a problem because evolution can hardly move from one ESS to another. If all hunters initially target the low‐value prey, mutants preferring the high‐value prey are counterselected by frequency‐dependent selection because they fail to coordinate with the majority. Hence, individuals are trapped in a suboptimal ESS and collective efficiency is not maximized.

All collective actions where individuals need to coordinate with one another, and where they can do so in either an efficient or an inefficient way, entail such an “evolutionary trap.” Inefficient coordinated behaviors evolve that cannot later be improved by natural selection because individual selection has no way of improving collective efficiency in a coordination game.

Evolutionary game theoreticians and evolutionary biologists have explored two hypotheses that can explain how this problem can be solved in nature. The first hypothesis is based on stochastic effects (Foster and Young 1990; Kandori et al. 1993; Young 1993; see also Nowak et al. 2004 in a different setting). In a finite population fixed in a particular ESS, counterselected mutants can rise in frequency due to genetic drift, and eventually destabilize the existing ESS, thereby moving the population away from the evolutionary trap, toward another, generally superior, ESS. The second hypothesis is based on group selection (Boyd and Richerson 1990, 2002; Richerson and Boyd 2005; Boyd and Richerson 2009). Due to chance, different groups of individuals may initially evolve different ESSes of the same game. If these groups compete with one another, the groups that happen to play the most efficient ESS will eventually prevail, allowing this strategy to spread in the entire population. In sum, according to available theories, coordination games suffer from an evolutionary trap problem, and collective efficiency in these games can ensue either from demographic stochasticity or from group selection, but not from plain individual selection.

However, so far, coordination games have been formally studied in models that were highly stylized, in particular with regard to the mechanistic underpinning of behavior, and these simplifications may have important consequences. In this paper, we describe simulations of a coordination game using evolutionary robotics (Nolfi and Floreano 2000; Doncieux et al. 2015). As compared to classic evolutionary game‐theoretical approaches, evolutionary robotics provides a more realistic modeling of individuals and their environment (Mitri et al. 2013; Trianni 2014), capturing in particular the practical problems raised by coordination  (Bernard et al. 2016).

In this setting, we show that the evolutionary trap actually disappears altogether. Simulated robots evolve a behavioral solution to coordinate with one another that generates indirect genetic effects (Wolf et al. 1998), hereby changing “the rules of the game.” Through their own behavior, robots transform a coordination game with multiple ESSes and an evolutionary trap problem into a simple individual optimization problem with a single, maximally efficient, ESS. In this transformed game, collective efficiency is reached by plain individual selection, with no need for genetic drift or group selection. We posit that many collective optimization problems may be solved in similar ways in nature.

## Results

We simulate a collective hunt in which two players must coordinate and attack together the same prey to gain a benefit. The two‐dimensional environment contains two types of prey, poorly nutritious prey called “boars” (worth 125 payoff units for each individual hunter), and highly nutritious prey called “stags” (worth 250 units for each hunter). Hunting alone is possible, but it provides 0 payoff unit (cf. payoff matrix in Table 1). This game features two strict Nash equilibriums (and thus two ESSes): to hunt either boars or stags, with the latter equilibrium providing a higher payoff. In technical terms, hunting stags is called the “payoff‐dominant” equilibrium.

**Table 1:** Possible outcome of a two‐player coordination game. The game features two hunters. Each player may choose to hunt either a “boar,” or a “stag.” This payoff matrix has two equilibriums, a suboptimal one (both players hunt the boar) and an optimal one (both players hunt the stag)

	Boar	Stag
Boar	125	0
Stag	0	250

The simulated robots we use as players are each driven by a multilayer perceptron (Rumelhart et al. 1986) that maps sensory inputs to motor outputs, with neural weights subject to artificial evolution. Each robot is endowed with proximity sensors all around its body. These sensors are capable, within a limited range, of discriminating between boars, stags, the other robot, and walls. So as to maintain the prey density constant, a captured boar (or stag) is removed from the environment and relocated to a new position (see Methods section).

In our simulations, we use an evolutionary algorithm often used in evolutionary robotics called ”elitist” selection (see Methods section). This algorithm reduces the effects of genetic drift while maintaining a small population size and therefore a reasonable computational cost. It consists in keeping, in each generation, the best *k* individuals from the previous generation while introducing *k* new mutants generated at random from these individuals. With k=1, this elitist algorithm actually consists in modeling a succession of replacements of a resident by a mutant (provided the mutant has higher fitness), as in standard evolutionary modeling (Eshel 1983; Geritz et al. 1998). Here, we consider an elitist algorithm with k=10 in order to capture the possible effects of genetic diversity.

### SELECTION CANNOT GENERATE COLLECTIVE EFFICIENCY IN A SIMPLE SETTING

The first question we address is whether the evolutionary transition from the least efficient to the most efficient ESS can occur.

First, we let 30 independent populations of simulated robots pre‐evolve for 3000 generations with only one boar and one stag, and modified payoff values: hunting stags temporarily yields no reward. We thus ensure that these 30 populations all evolve the boar‐hunting equilibrium, with all individuals always targeting boars and avoiding stags.

**Figure 1:**
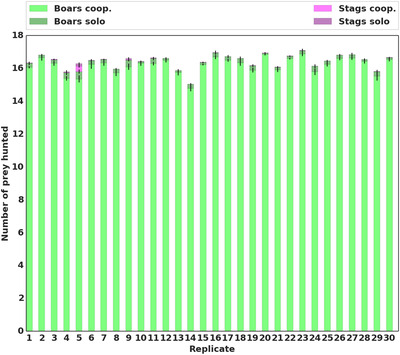
Mean proportion of prey hunted. Repartition of the prey hunted by the best individual in each replicate, at the last generation of evolution (generation 6000). We differentiated between the type of prey hunted (boar or stag). Rewards were 125 for a boar and 250 for a stag (Table 1).

Second, each of these 30 populations of evolved boar hunters is used as the seed for another 6000 generations of evolution, with the regular rewards for each prey reinstated (Table 1). In spite of its collective superiority, stag hunting never evolves within the next 6000 generations for any of 30 independent replicates (Figure 1). In every replicate, the mean proportion of stags hunted remains at 0 throughout the 6000 generations. Hence, individuals are genuinely trapped in the suboptimal equilibrium. That is, collective efficiency cannot ensue from plain individual selection.

### GENETIC DRIFT ALLOWS TRANSITION TO THE EFFICIENT EQUILIBRIUM

**Figure 2:**
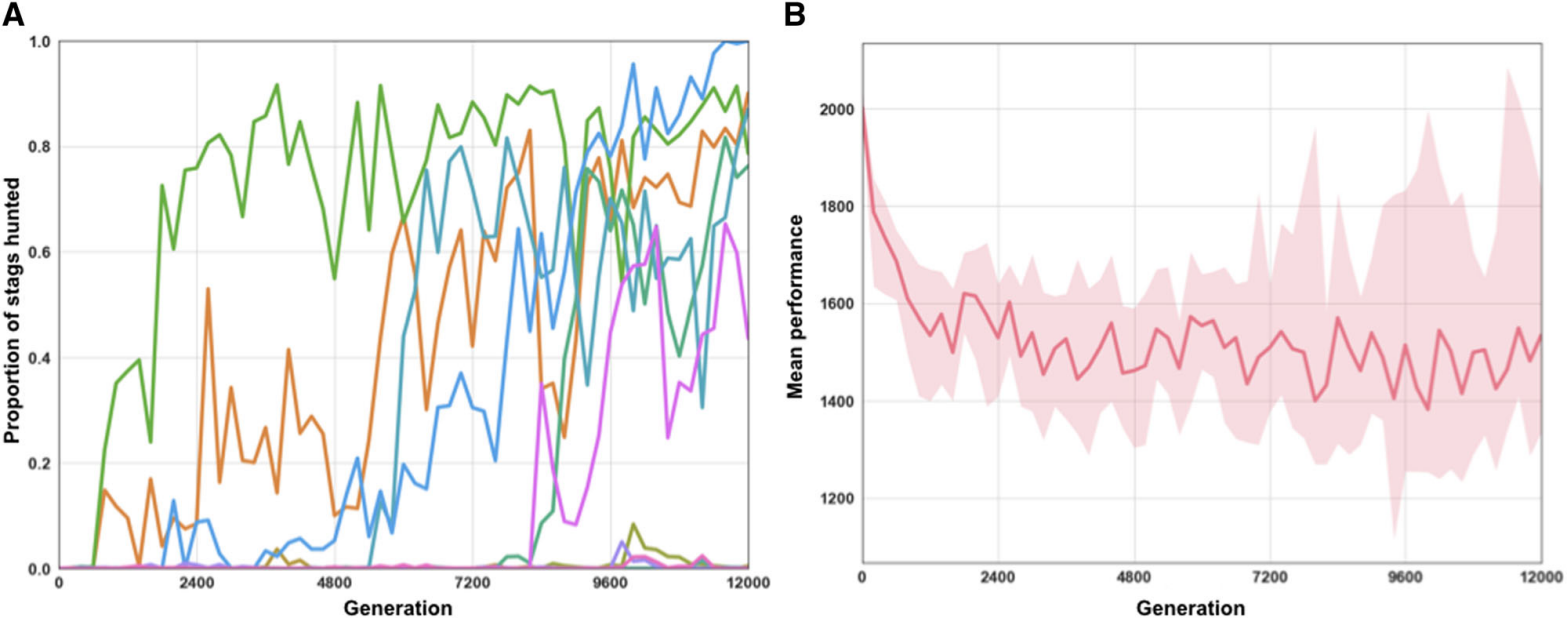
Evolution of the type of prey hunted and average reward under the fitness‐proportionate algorithm (i.e. with strong genetic drift). (A) Proportion of each type of prey hunted in every replicate throughout evolution. (B) Average reward of the best individual in each of the 12 replicates.

The elitist selection algorithm used in our simulations has the advantage of significantly reducing the effects of genetic drift while maintaining a reasonable computational cost. It is therefore only natural to observe that the transition from one equilibrium to another does not occur in our simulations, because this transition requires the intervention of demographic stochasticity (Foster and Young 1990; Kandori et al. 1993; Young 1993). In order to find out whether demographic stochasticity allows this transition to take place in our setting, we carry out the same simulations with a different selection algorithm. We consider a population of 20 individuals in which individuals simply reproduce in proportion to their fitness according to a Wright–Fisher model (also called a “fitness‐proportionate” algorithm in evolutionary robotics). We initialize 12 replicates with a genotype pre‐evolved under the elitist algorithm and playing the boar‐hunting strategy, and let them evolve for 12, 000 generations under the fitness‐proportionate algorithm. Among these 12 replicates, we observe that the frequency of stag hunting increases above 50% in about half of the cases (Figure 2A), but we observe that they mostly play a mixed strategy (they hunt the stag with a probability <1) and that their performance is reduced as compared to the initial genotype (Figure 2B), which can be understood as a consequence of the fact that genetic drift leads to the fixation of deleterious mutations.

In accordance with theoretical predictions, therefore, demographic stochasticity makes it possible to escape from an evolutionary trap and reach a more efficient equilibrium. It may also be noted that the stochastic transition away from the trap would be even easier in a genetically structured population, in which individuals would interact with relatives, because this would increase the probability that two mutants play together and successfully coordinate on the stag (Skyrms 2004), but we have not tested this possibility.

We will now show, however, that in practice genetic drift is not necessary to get out of an evolutionary trap because there is often no evolutionary trap at all. For this, in the remaining of the article, we exclusively use the elitist selection algorithm in which genetic drift is low and the transition to stag hunting impossible in the simple setting.

### COLLECTIVE EFFICIENCY CAN BE ACHIEVED BY SELECTION, IN A MORE COMPLEX SETTING

In practice, predators are unlikely to live in a world with a single prey of each kind. In a realistic environment, hunters must agree on a specific *“*individual” prey to hunt (Alvard and Nolin 2002; Alvard 2003; Drea and Carter 2009), and not just on the *“*type” of prey.

To investigate the consequences of this complication, we follow the same procedure as before. We pre‐evolve 30 independent populations of pure boar hunters, using modified payoff values, with the stags never bringing any reward, but this time in an environment with several (9) identical boars and several (9) identical stags present.

We then let each of these 30 populations evolve for another 6000 generations with regular payoff values (Table 1) in the same environment with several boars and stags present (see Methods section).

**Figure 3:**
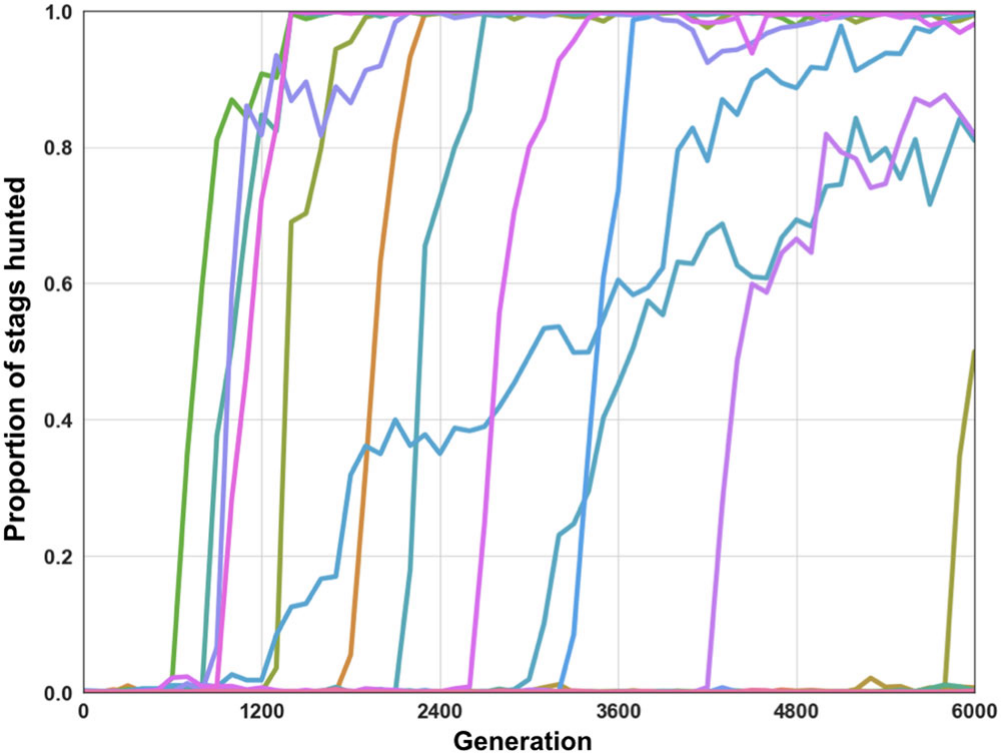
Evolution of the type of prey hunted. Proportion of each type of prey hunted in every replicate throughout evolution in the “complex environment” setting. Rewards were 125 for a boar and 250 for a stag (Table 1).

In this setting, we observe that the transition from boar hunting to stag hunting does occur in 12 replicates out of 30 (Figures 3 and 5). This significantly differs from the previous results obtained in a simpler environment (One‐tailed Mann–Whitney *U* test on the number of replicates where the transition happened *P*‐value < 0.0001). Environmental complexity promotes the evolutionary transition toward the payoff dominant equilibrium in 40% of the replicates.

**Figure 4:**
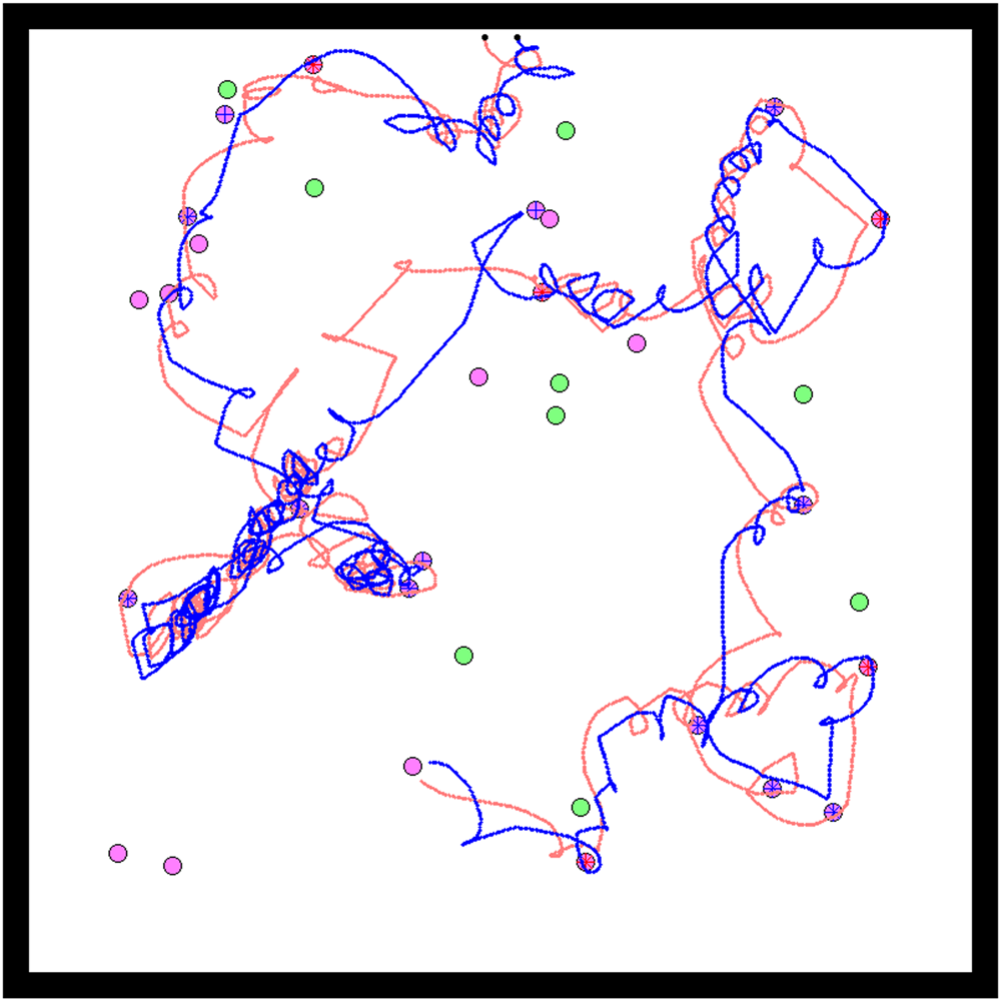
Example of the turning strategy. Example of a simulation where both individuals adopted a “turning” strategy. The path of the agents during this simulation is represented in red and blue, starting from their initial positions (represented by black dots). Each disc represents a prey in the environment. Boars are represented in green and stags in purple. When a prey was killed cooperatively, a red cross (respectively blue) is shown on the prey if the red agent (respectively blue) arrived at this prey first.

Taking a closer look at these 12 “successful” replicates reveals a particular kind of coordination strategy for collective hunting. Because the environment is more complex, individuals need to react to each other's behavior to stay together and converge on the same prey. To this end they evolve a behavioral strategy, which we refer to as the “turning” strategy, whereby they constantly turn around one another. This strategy ensures that they keep their partner in their line of sight and move toward a prey at the same time. Due to their proximity, an individual who gets on a prey is likely to be joined quickly by their partner (Figure 4; a video of this strategy is also available in Supporting Information).

This behavioral strategy has an evolutionary implication. Because hunters react to each other's behavior, a mutation affecting the behavior of one individual can also modify the behavior of her partner. A mutant attracted to stags rather than boars may thus succeed in hunting by transforming, albeit temporarily, her partner into a stag hunter as well. As a result, the evolutionary transition away from the suboptimal trap is facilitated in comparison with the simple environment.

### DIVISION OF LABOR FURTHER FACILITATES COLLECTIVE OPTIMIZATION

The turning strategy obtained so far results from both hunters demonstrating identical behaviors, whether related to moving together or selecting a prey. While this similarity in behavior makes it possible for hunters to coordinate toward hunting the same prey, both individuals spend a significant amount of time turning around one another. This results in a tedious process of targeting a particular prey. The question is open as to the existence of more efficient coordination patterns, in particular with respect to assuming complementary behavioral strategies.

We posit that one possible limitation is the lack of expressivity of our choice of control function, a limitation that may not exist in nature. Although multilayered perceptrons are theoretically universal approximators, this is hardly the case in practice (Cybenko 1989). In particular, the ability to switch from one behavioral pattern to a completely different one may be required for breaking behavioral symmetry between two individuals, but may be hindered by the limitation of the controller used thus far (see Ferrante et al. 2015 and Waibel et al. 2009).

To explore the possible benefits of more complex decision‐making capabilities, we enable each robot with the possibility to choose from two controllers, depending on the context at hand. To do so, we introduce a new evolutionary operator: the “network duplication” operator. Loosely inspired by gene duplication (Hughes 2002), a newly created individual may be subject to the complete duplication of its artificial neural network. Conversely, an individual with two networks may have one deleted. In this setup, any individual may possess either one or two network(s). Initially, after a duplication event, an individual has two identical networks (like two genes after a genetic duplication). But, just like duplicated genes in different loci in the genome, these two networks each have a different “place” in the individual's “genome.” The individual thus has a “network 1” and a “network 2” (even if they are identical). And these two networks then evolve independently of each other, under the effect of mutation and selection. Importantly, whenever two individuals who possess two networks each interact with one another, we ensure that each of them expresses a different copy of their own networks (see Methods section). That is, we assume that one individual expresses his “network 1” while the other expresses his “network 2” and this regardless of whether these two networks are identical or very different from one another.

We use the same simulation procedure as before: 30 populations are pre‐evolved independently where only boar hunting yields a reward, and this time we introduce network duplication. We observe a significant difference with respect to previous simulations, as in all 30 replicates, we observe the evolution of asymmetrical hunting behaviors in the form of a “leader–follower” division of labor.

**Figure 5:**
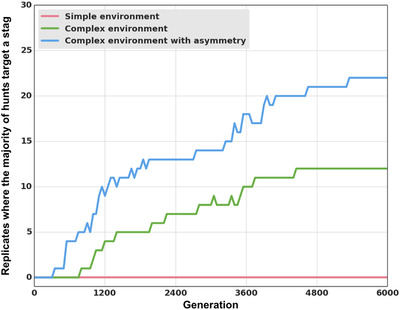
Number of stag‐hunting replicates Number of replicates (out of a total of 30) where cooperative stag hunting accounted for the majority of hunts (i.e. more than 50% of the prey hunted were stags hunted cooperatively) in the “simple environment,” “complex environment,” and “complex environment with asymmetry” settings. The simple environment and complex environment settings are the same as previously shown (Figure 3). In the complex environment with asymmetry, duplication of the neural network could occur.

**Figure 6:**
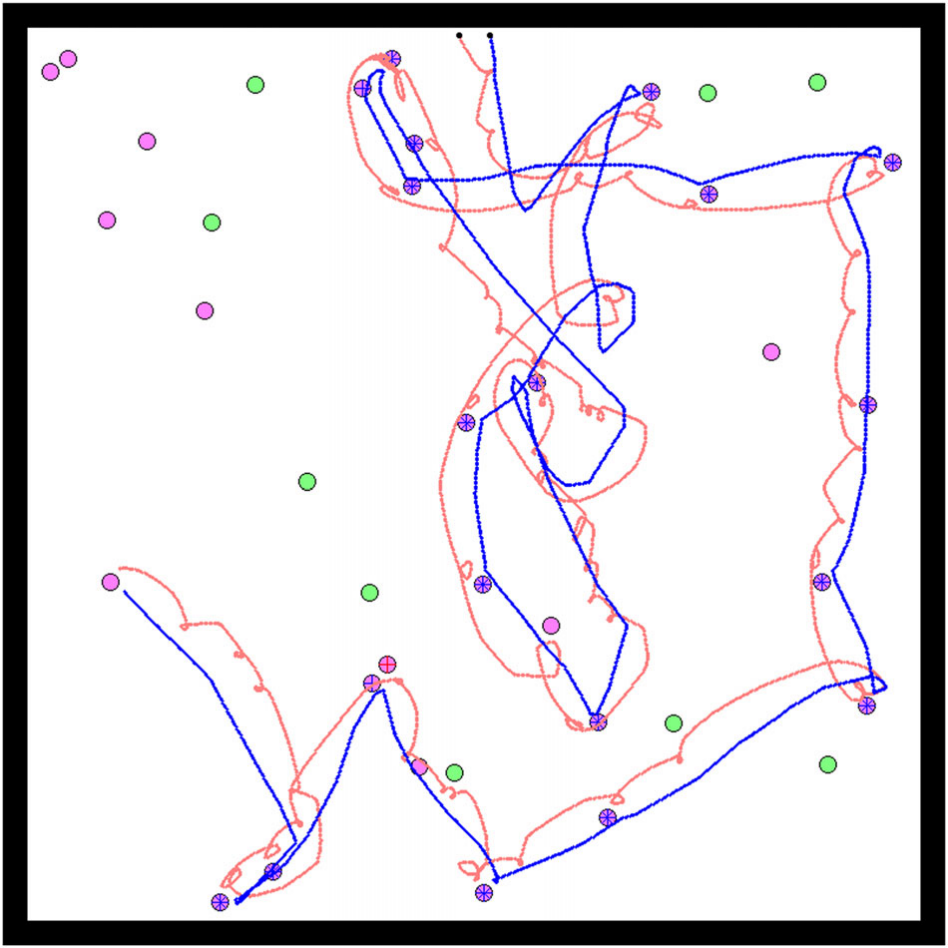
Example of the leader–follower strategy. Example of a simulation where both individuals adopted a leader–follower strategy. The path of the agents during this simulation is represented in red and blue, starting from their initial positions (represented by black dots). Each disc represents a prey in the environment. Boars are represented in green and stags in purple. When a prey was killed cooperatively, a red cross (respectively blue) is shown on the prey if the red agent (respectively blue) arrived on this prey first.

Results are also significantly different than in both previous treatments when the original payoff matrix is reinstated (Table 1). The transition to stag hunting occurs in 22 replicates out of 30 (Figure 5), as compared to 0 in the simple environment, and 12 in the complex environment without network duplication (one‐tailed Mann–Whitney *U* test on the number of replicates where the transition happened: *P*‐value < 0.0001 and *P*‐value = 0.015, respectively). As in the pre‐evolution runs, the “leader” guides the pair toward a given prey, always arriving first, while the “follower” keeps the leader in its line of sight at all times and joins her afterward on the prey (Figure 6; a video of this strategy is also available in Supporting Information).

This cognitive division of labor stems directly from the duplication of the neural controller: once duplicated, one version of the network always ends up encoding for the leader behavior, while the other encodes for the follower behavior, just like duplicated genes of the same family encode slightly different functions.

**Figure 7:**
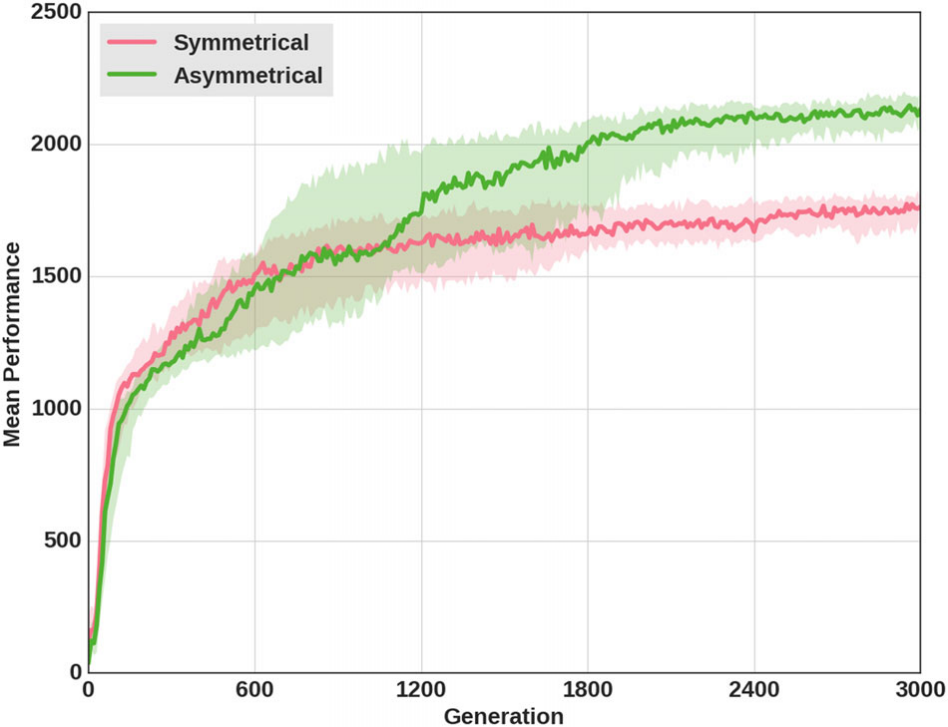
Average reward of the turning strategy and the leader–follower strategy. Average reward of the best individual in each of the 30 replicates under the symmetrical (turning) or asymmetrical coordination strategy (leader–follower). Rewards were 125 for a boar and 250 for a stag (Table 1).

The division of labor has two consequences. First, it improves hunting efficiency. In the turning strategy, the symmetry of decision‐making sometimes hinders the ability to reach a consensus. Even though turning promotes coordination, individuals often still fail to converge on the same prey. In comparison, performance is significantly higher in the leader–follower strategy (Figure 7, one‐tailed Mann–Whitney *U* test on the mean reward at last generation, *P*‐value < 0.001): the frequency of coordination failures is reduced thanks to a clear separation of roles.

Second, the division of labor has an evolutionary consequence. In the leader–follower strategy, just as in the turning strategy, hunters react to each other's behavior and are therefore also prone to react to mutants' behavior. But, in contrast to the turning strategy, this response is asymmetrical and, therefore, more precise. Any mutation affecting the leader's behavior also changes completely the behavior of the follower. That is, a mutation in a single individual automatically affects two individuals at the same time. As a consequence, the adaptive valley between boar hunting and stag hunting disappears or, put differently, boar hunting ceases to be an equilibrium. Any increase in the probability of hunting a stag rather than a boar, when playing the role of a leader, is directly favored by individual selection, and pure stag hunting therefore becomes the only evolutionary equilibrium.

**Figure 8:**
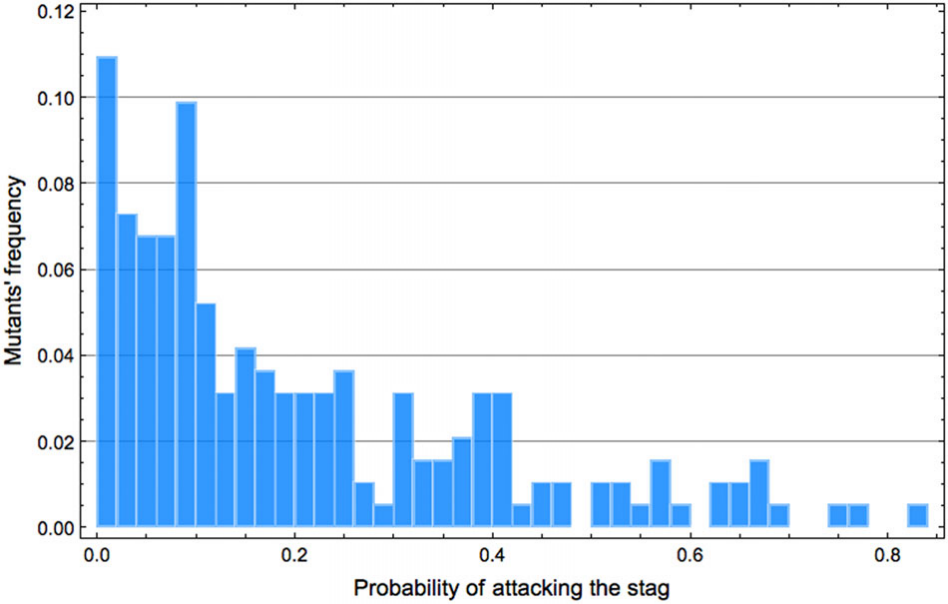
Probability to prefer stags over boars in mutants generated randomly from a pure boar‐hunter genotype. Among the 10^5^ mutants generated, we display only the 192 mutants whose probability to hunt stags is greater than 1% (see Methods section).

In fact, behavioral coordination between players (whether leader–follower or turner) facilitates the evolutionary transition also for another slightly different reason. In a robotic setting like this one, stag hunters must appear by random changes in the connection weights of boar hunters' neural networks, and multiple such changes separate a pure and well‐optimized boar hunter from a pure and well‐optimized stag hunter. As a result, mutants playing the stag‐hunt strategy are extremely rare in a population of pure boar hunters. This is confirmed in supplementary simulations (see Figure 8, and Methods section), where we analyzed the behavior of 10^5^ mutants generated randomly from a pure and well‐optimized “boar hunter” genotype. This analysis showed that, at best, the random mutants merely had a probabilistic tendency to target the stag. That is, they played a “mixed” rather than a pure strategy. In the absence of a coordination mechanism, these intermediate mutants are strongly counterselected as their mixed strategy generates many coordination failures (if two players both play stag with probability *P*, they fail to coordinate with probability 2P(1−P)). Hence, these intermediate mutants cannot bring about the transition to stag hunting. On the contrary, if players are able to coordinate with one another, they can avoid all coordination failures. For example, in the case of a leader–follower, if the leader plays the mixed strategy that consists in choosing the stag with probability *P* then the two individuals will successfully hunt stag with probability *P* and boar with the complementary probability. That is, they never fail to coordinate. As a result, intermediate strategies can provide a possible evolutionary pathway between pure boar hunting and pure stag hunting.

## Discussion

Collective actions often require several individuals to make coordinated choices. As a result, their efficiency, or lack of efficiency, is a collective property, not a property of any particular individual. This raises an evolutionary difficulty because natural selection acts on individual, not collective, properties. Collective actions are thus subject to an “evolutionary trap” problem. Once a relatively successful but still perfectible collective organization has evolved, any single mutant playing a better strategy will be counterselected due to her lack of coordination with others. For collective efficiency to be reached by evolution, several individuals would all somehow have to “mutate collectively,” but genetic mutations do not occur in several organisms at the same time.

In this paper, we studied this problem in artificial robotics simulations. We simulated the life and the long‐term evolution of a population of simple robots that played a 2 × 2 coordination game. Robots were hunters who could choose between two types of prey that were either poorly nutritious or highly nutritious. But they could only be successful if they converged together on the same prey. Hence, they faced a coordination problem with two ESSes—hunting poorly nutritious prey or hunting highly nutritious prey, and an adaptive valley in between. Our aim was to find out how the evolutionary trap problem materializes and how it is solved—or not solved—in a model possessing a greater degree of realism than conventional models of coordination games.

We first confirmed the existence of an evolutionary trap. In a simple setting where the environment was constituted of two individual prey only—one poorly nutritious and one highly nutritious—if we initially forced the simulated robots to play the suboptimal ESS (attacking the poorly nutritious prey), all populations of robots remained stuck in this ESS “forever,” that is, at least for the 6000 generations of our simulations. Individual mutants who were targeting the better prey could not be favored due to their singularity.

However, we then showed that this problem actually disappears in a more realistic setting. In a richer environment constituted of several prey of each kind—several poorly nutritious prey and several highly nutritious prey—individuals needed to actively coordinate with their partner to converge on the same prey. To resolve this problem, they evolved behavioral tactics to keep track of and follow their partner. In our simulations, we observed the evolution of two such tactics. In the first series of simulations, individuals constantly turned around one another, never moving away from their partner, which increased the probability that they both would eventually converge on a prey. In other simulations where we authorized a behavioral asymmetry between partners, individuals evolved a leader–follower strategy whereby a single individual chose a prey, whereas the other simply followed her.

These coordination strategies evolved because they had immediate individual benefits. They increased the probability for individuals to hunt successfully. But they also had an unintended evolutionary consequence. When individuals had the capacity to coordinate with each other, a mutation affecting the behavior of one individual also indirectly modified, phenotypically, the behavior of his/her partner, almost as if individuals had mutated “collectively.” In quantitative genetics, such an effect is called an “indirect genetic effect” (Wolf et al. 1998) because a gene affects the phenotype of an individual in which it is not directly expressed. Indirect genetic effects are well known for changing the evolutionary process in sometimes dramatic ways, by altering the genotype‐phenotype relationship. In the present case, behavioral coordination tactics evolved to deal with the uncertainty of the “normal” environment in which one's partners only targeted suboptimal prey but the precise individual prey they were targeting could vary, which required being able to follow them. However, once evolved, coordination tactics also happened to work efficiently when interacting with mutants who preferentially targeted other types of prey. They led one to follow and coordinate with mutants like they did with “normal” residents. Consequently, genetic mutants that preferred targeting the most nutritious type of prey were directly favored by individual selection because they always had a resident who accepted to follow them since she was indirectly influenced by their mutated gene. The suboptimal coordination equilibrium was no longer an evolutionary trap. In finding a solution to the “behavioral” coordination problem, individuals solved the “evolutionary” coordination problem as well.

Put another way, behavioral coordination strategies changed the nature of the game. Individuals initially played a coordination game in which two players needed to jointly evolve a compatible preference. This raised a bootstrapping problem and made the transition from one equilibrium to another unlikely. By evolving endogenously a coordination strategy, individuals turned this game into a plain optimization game in which a single player was simply selected to choose the best possible prey.

Beyond the particular setting considered in this paper, we think these results reveal a general principle that could play a role in all games with multiple equilibriums, that is, in all coordination games, but also in repeated games such as the repeated prisoner's dilemma (see, for instance, Aumann and Shapley 1994; Boyd 2006). As a rule, there are many reasons why the behavior of one's partners will vary in all these games, making it necessary for one to adapt plastically to this variability (Mcnamara et al. 2010). In our simulations, for instance, individuals evolved a coordination strategy to adapt to the precise location where their partner was heading, but the same principle should hold in other settings as well. Even though behavioral plasticity originally evolves merely to deal with partners' phenotypic variability, it also happens to generate an adaptive response in front of mutants. These mutants probably did not exist when behavioral plasticity evolved, but they nevertheless happen to trigger the exact same response. And because this response was originally meant to maximize efficiency, it is likely to do so with mutants, too, as our simulations illustrate. Hence, there is a general reason why the plastic response of individuals to each other should often “change the rules of the game” and smoothen the transition to efficient collective behaviors.

## Methods

### SIMULATION SETUP

The environment is a 800 by 800 unit arena with four solid walls. Each simulation is conducted with a pair of hunters (the robotic agents) and a varying number of prey of two types, boars and stags with respective rewards 125 and 250 (Table 1). The initial positions of the prey are random and the prey cannot move. To capture a prey, the two hunters have to stay in contact with it for 800 time steps (out of a total of 20, 000 time steps for each simulation). Both robots have to be in contact with the prey at the end of the 800 time steps for the hunt to be considered successful. Once captured, the prey is removed and replaced at a random position in the arena. In the “simple” environment condition, there is always exactly one boar and one stag present in the environment. In the “complex” environment condition, there is always nine prey of each type. Robots (that is, hunters) begin the simulation next to each other at the top of the arena and can then move freely in the environment. To do so, they are equipped with a set of sensors and two independent wheels connected by a fully connected multilayer perceptron. Sensors comprise 12 proximity sensors and a camera. Proximity sensors are evenly distributed around the robot's body, and each has a range of 40 units. A proximity sensor is a ray toward a particular direction indicating to the robot the distance of the first obstacle in this direction. The camera is placed on the front of the robot, and its 90○ field of view is divided into 12 equally spaced rays. Each ray of the camera indicates the type (that is, hunter, boar, or stag) and the proximity of the nearest agent in its direction. Robots are individually controlled by a fully connected multilayer perceptron with a single hidden layer. The inputs of the neural network are fed with the sensory data of the robot. One input neuron is used for each of the 12 proximity sensors, with maximal (respectively minimal) neural activity when the agent is directly in contact with an obstacle (resp. when there is no obstacle in the range of the sensor). Three neurons are used for each of the 12 rays of the camera: two neurons to encode the type of obstacle in a two‐bit binary value and one neuron to encode the proximity of the obstacle. Finally, there is a bias neuron whose value is always equal to one. The total number of input neurons is 49. The hidden layer contains eight neurons, while the output layer contains two neurons. These two output neurons control the speed of the left and right wheels; minimal (respectively maximal) activity results in maximal backward (respectively forward) actuation. The activation function used to compute outputs is a sigmoid function. Connection weights are each encoded in a single gene (the total genome size is 410).

### SIMULATING ARTIFICIAL EVOLUTION

In each of the 30 independent replicates, we let a population of 20 individuals evolve. Each individual is encoded as a genome, where each gene codes for a connection weight of the multilayer perceptron controller. Every gene in the genome is first initialized with a random value sampled uniformly in [0, 1]. In each generation, the performance of every individual is evaluated by matching her with five different random partners. In turn, the performance of each pair of partners is evaluated through five independent trials. Hence, the fitness of every individual is computed in each generation as an average across 25 independent trials. We then apply a (10 + 10) elitist selection algorithm  (Goldberg 1989). That is, generation t+1 is composed of the 10 best individuals of generation *t* plus 10 mutants generated from a single parent of generation *t*. Mutations are sampled according to a Gaussian operator, with a SD of $2\times 10^{-1}$ and a per‐gene mutation probability of $5\times 10^{-3}$.

### DUPLICATION AND COEVOLUTION OF NEURAL NETWORKS

To study the effect of an asymmetry between hunters, we allow the duplication of neural networks. Every individual initially has a single neural network but duplication and deletion events can occur randomly (at the same moment of the life cycle than mutation). When duplication occurs, each gene is duplicated to create a new genome encoding for a second neural network that can then evolve independently of the first. When deletion occurs, one of the two neural networks of the individual is deleted randomly. Duplication occurs with a probability $5\times 10^{-2}$ and deletion with a probability $5\times 10^{-3}$ per generation.

### ANALYSES OF BOAR–HUNTER MUTANTS WITH RESPECT TO STAG HUNTING

We generate 100, 000 random mutants from a well‐optimized boar–hunter genotype (with the same mutation parameters than in our evolutionary simulations), and assess each mutant's hunting preferences. From these 100, 000 mutants, we extract 192 mutants that displayed a *P* > 0.01 of hunting the stag. Figure 8 shows the distribution of the preferences of these 192 mutants. Most mutants have only a small probability to hunt stags. In particular, not a single pure stag hunter can be found among the 100, 000 mutants.

Associate Editor: A. Gardner

## Supporting information


**Data video S1**.

**Data video S2**.
